# Modeling the human placental barrier to understand *Toxoplasma gondii*´s vertical transmission

**DOI:** 10.3389/fcimb.2023.1130901

**Published:** 2023-03-09

**Authors:** Paula Faral-Tello, Romina Pagotto, Mariela Bollati-Fogolín, Maria E. Francia

**Affiliations:** ^1^Laboratory of Apicomplexan Biology, Institut Pasteur de Montevideo, Montevideo, Uruguay; ^2^Cell Biology Unit, Institut Pasteur de Montevideo, Montevideo, Uruguay; ^3^Departamento de Parasitología y Micología, Facultad de Medicina, Universidad de la República, Montevideo, Uruguay

**Keywords:** Toxoplasma gondii, human placenta, trophoblast, maternal-fetal interface, vertical transmission, *in vitro* models

## Abstract

*Toxoplasma gondii* is a ubiquitous apicomplexan parasite that can infect virtually any warm-blooded animal. Acquired infection during pregnancy and the placental breach, is at the core of the most devastating consequences of toxoplasmosis. *T. gondii* can severely impact the pregnancy’s outcome causing miscarriages, stillbirths, premature births, babies with hydrocephalus, microcephaly or intellectual disability, and other later onset neurological, ophthalmological or auditory diseases. To tackle *T. gondii’s* vertical transmission, it is important to understand the mechanisms underlying host-parasite interactions at the maternal-fetal interface. Nonetheless, the complexity of the human placenta and the ethical concerns associated with its study, have narrowed the modeling of parasite vertical transmission to animal models, encompassing several unavoidable experimental limitations. Some of these difficulties have been overcome by the development of different human cell lines and a variety of primary cultures obtained from human placentas. These cellular models, though extremely valuable, have limited ability to recreate what happens *in vivo*. During the last decades, the development of new biomaterials and the increase in stem cell knowledge have led to the generation of more physiologically relevant *in vitro* models. These cell cultures incorporate new dimensions and cellular diversity, emerging as promising tools for unraveling the poorly understood *T. gondii*´s infection mechanisms during pregnancy. Herein, we review the state of the art of 2D and 3D cultures to approach the biology of *T. gondii* pertaining to vertical transmission, highlighting the challenges and experimental opportunities of these up-and-coming experimental platforms.

## 1 Introduction

*Toxoplasma gondii* is an ubiquitous apicomplexan parasite that can infect virtually any warm-blooded animal, and has the ability to access and infect immune-privileged sites such as the brain, the eye and the placenta. The parasite is transmitted among animals by ingestion of persistent cysts lodged in the brain or skeletal muscle. When a felid consumes chronically infected tissues with bradyzoite, the parasite can initiate its sexual differentiation cycle within its intestinal epithelium. Gametes can sexually recombine which will eventually lead to shedding of unsporulated oocysts. Upon contact with oxygen, oocysts will sporulate and lead to infective environmentally resistant oocysts (Ferguson, 2002) that can be consumed by intermediate hosts, including pregnant women. Altogether, these characteristics make *T. gondii* one of the most successful zoonotic parasites worldwide (Flegr et al., 2014).

Acquired infection during pregnancy and placental breach is at the core of the most devastating consequences of toxoplasmosis. *T. gondii* can severely impact the pregnancy’s outcome causing miscarriages, stillbirths, premature birth, babies born with conditions such as hydrocephalus, microcephaly or intellectual disability, and other later onset neurological, ophthalmological or auditory diseases (Torgerson and Mastroiacovo, 2013). Clinical manifestations may vary depending on gestation period, fetal size, inoculum, and genetic background of the triad: mother, fetus and parasite (Dubey et al., 2021). In humans, it is well established that the outcome is dependent on the trimester of gestation. Infections in early pregnancy are often associated with pregnancy loss (Dubey et al., 2021), while mid gestation and third trimester infections are more frequent and often result in fetal malformation (Desmonts and Couvreur, 1974a; Desmonts and Couvreur, 1974b).

It has been observed that congenital toxoplasmosis is more frequent when acute infection occurs during the second half of pregnancy, particularly the third trimester where placental layers separating maternal blood from fetal blood are thinner (Błaszkowska and Góralska, 2014) and blood flow increases substantially. However, these observations must be analyzed considering the generalized worldwide sub-diagnosis of toxoplasmosis (Nayeri et al., 2020), and that the etiology behind most spontaneous abortions (first trimester) remain undetermined, among which *T. gondii* should not be ruled out (Nayeri et al., 2020). Moreover, latent infection is highly prevalent (Rostami et al., 2020) and is responsible for many neuropathological effects, pre-eclampsia, thyroid diseases and infertility, among others (Rostami et al., 2016). Although the associations between latent infection and different gestational outcomes are still under active debate (Mocanu et al., 2022), there is evidence of association with slower fetal development and slower acquisition of postnatal motor skills (Kaňková and Flegr, 2007; Kaňková et al., 2012). On the other hand, in those countries that include screening tests in routine prenatal care schemes, opportune treatment can impact differently vertical transmission rates between first and third-trimester congenital infections.

It has long been accepted that chronic infections prevent reinfections and protect the fetus from vertical transmission. However, this paradigm has recently been challenged, as growing evidence suggests that reinfection is possible when a genetically distinct strain reinfects a seemingly “immunized” individual (Elbez-Rubinstein et al., 2009; Jensen et al., 2015). This is important since different strains circulate worldwide, particularly in South America where there is a predominance of atypical strains (Galal et al., 2019).

The host’s proper modulation of her immunity during the course of gestation is paramount to its maintenance and to a healthy outcome. Thus, interfering with parasite-specific factors would be the safest intervention strategy in the context of pregnancy. However, their involvement in vertical transmission still remains unclear. In fact, except for a handful of exceptions, the parasite factors licensing vertical transmission remain virtually unidentified (Arranz-Solís et al., 2021).The role of the immune system in protecting the fetus against *T. gondii* has been exhaustively studied, and specific alleles in immune response-related genes that might favor or prevent vertical transmission have been described (Reviewed in (Ortiz-Alegría et al., 2010). However, the host’s immune system has also been shown to be the target of parasite-specific factors which by way of modulating cellular mobility, use them as trojan horses for dissemination (Ortiz-Alegría et al., 2010). Three secreted parasite factors, TgWIP, Tg14-3-3 and ROP17, have been shown to generate hypermobility of dendritic cells, monocytes and natural killer cells which the parasite uses to reach immune-privileged sites (Arranz-Solís et al., 2021). CCL22 is a chemokine which plays critical roles in immune-tolerance. GRA28 is a dense-granule secreted protein that modulates the secretion of CCL22 in the host infected cells, including placental cells. Parasites lacking GRA28 are not able to disseminate (Rudzki et al., 2021). GRA28 was also recently shown to impact infected macrophage mobility by inducing a dendritic cell like behavior, caused by the transcriptional rewiring of the infected cell (Hoeve et al., 2022).

*In vitro* modeling of the life stages of *T. gondii* has been traditionally limited to 2D cultures whereby the fast growing tachyzoite form of the parasite expands quickly and efficiently, allowing for the generation of large amounts of material for different analyses. Albeit *in vitro* bradyzoites do not bear an absolute biological resemblance to their *in vivo* counterparts, the partial access to their biology offered by *in vitro* models has greatly contributed to our understanding of the chronic forms of parasite persistence (Mayoral et al., 2020).

In stark contrast, the interplay among tachyzoites, bradyzoites and host factors, in the context of transplacental transmission cannot thus far be mimicked in traditional 2D cultures. The study of these aspects of parasite biology has thus far relied on animal models, encompassing several unavoidable experimental limitations. Nonetheless, recent technological breakthroughs in 3D and 2D culture systems provide promising routes for exploring aspects of parasitic life traditionally inaccessible. Herein, we review the state of the art of 3D and 2D cultures to approach one of the most poorly understood aspects of the biology of *T. gondii*, highlighting the challenges and experimental opportunities of these up-and-coming experimental platforms.

## 2 Placental architecture

**Figure 1:**
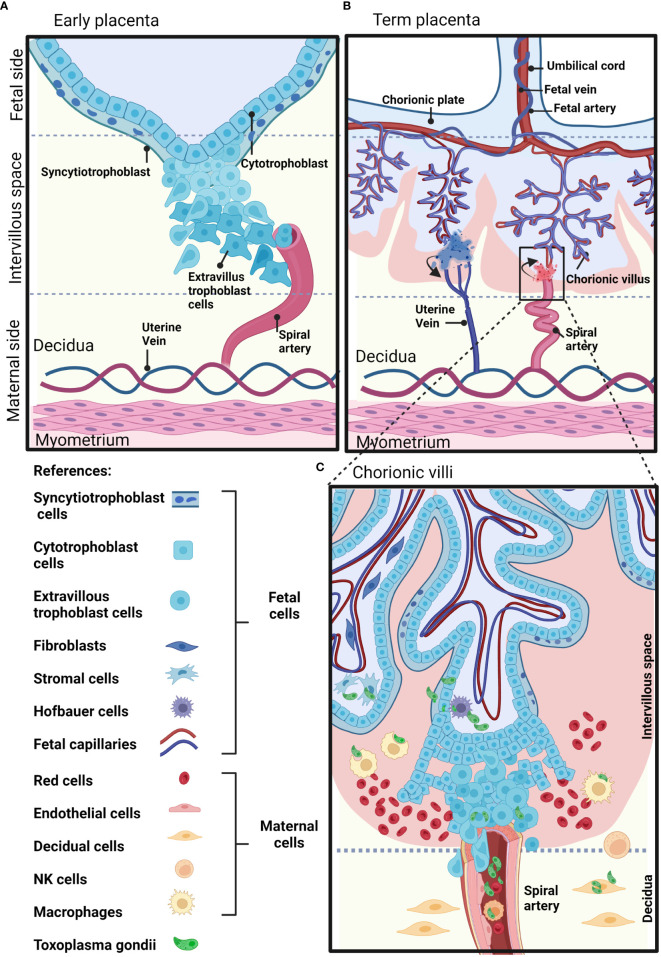
Schematic representation of a human placenta at two developmental time points. **(A)** Early placenta. The cytotrophoblasts fuse together and form the polynucleated syncytiotrophoblast layer, or differentiate into invasive extravillous trophoblasts. Extravillous trophoblasts invade the maternal decidua, and remodel the maternal arteries. **(B)** Term placenta. The fetal part of the fetal-maternal interface consists of chorionic villi that extend from the chorionic plate into the intervillous space and bathe in maternal blood. On the maternal side, the decidua is in direct contact with fetal membranes and the invading fetal extravillous trophoblasts. The maternal blood enters the intervillous space through spiral arteries and leaves this compartment through uterine veins. **(C)** The inset shows representative maternal and fetal cell types on a longitudinal section of a human-term placenta. Parasite structures indicating sites susceptible to *T. gondii* infection are shown in green. Created with BioRender.com.

The placenta is a temporary fetal-maternal organ responsible for most communications between mother and fetus. It is formed during embryo implantation at the place where fetal membranes contact the surface of the epithelium of the uterine mucosa (Moore et al., 2019). The placenta is a very divergent organ that varies among different species regarding its exterior form, the number of membranes, vascular arrangement and the number of tissues separating fetal blood from maternal blood (Furukawa et al., 2014). The human placenta is hemochorial, meaning that vascularized chorionic villi (fetal portion) float freely fully bathed in maternal blood. This close proximity is the result of an active and deep invasion process led by a specific type of embryonic tissue called trophoblast (TB). TB forms early after fertilization in the morula stage (12-32 cells zygote) and will differentiate into cell subtypes according to location and function. Cytotrophoblast cells (CTB) consist of flattened cells surrounding the blastocyst and will form the fetal part of the placenta (Moore et al., 2019). CTB forms a layer of mononucleated cells that are mitotically active and give rise to the syncytiotrophoblast (STB), a rapidly expanding increasing mass of fused cells where no cell boundaries are observable (Moore et al., 2019). Until week 20, fetal villi are covered through all their extension with CTB and STB and after the 20th week, CTB disappears over large areas leaving only STB to stand between maternal blood and fetal endothelium (**Figures 1A, B**). CTB subtypes are extravillous TB (EVT) that abandon the fetal villi margins to migrate towards the decidua and forms a column that anchors to the decidua, and endovascular CTB, which migrates and colonizes spiral arteries regulating the vascular remodeling that is needed to secure blood flow (Pollheimer et al., 2014). The mentioned cell types are highlighted in **Figure 1C**.

How *T. gondii* crosses the placental barrier, infects the trophoblast, reaches fetal vascularity and disseminates, remains virtually unknown. This is partially owed to the difficulties and complexity of accurate placental human models and the ethical concerns associated with using human-derived samples. Nevertheless, from infections on model animals and different cell types, including human placenta-derived models, a number of mechanisms have been proposed. These include: 1) Infection of the maternal decidua and immune decidual cells which includes the trojan horse strategy; 2) Infection of EVT, fetal cells that deeply invade maternal endometrium; 3) Direct molecular adhesion of parasites to STB; 4) Active degradation of extracellular matrix (ECM) and 5) Infection as a consequence of inflammation-induced tissue damage. These alternatives are exhaustively reviewed in (Megli and Coyne, 2021; Rojas-Pirela et al., 2021).

In terms of temporal development and placental architecture, two scenarios can be identified that represent moments of particular vulnerability for vertical transmission. As mentioned, fetal trophoblast invades maternal decidua as deep as to encounter spiral arteries during the first trimester. This creates a scenario in which parasites present in maternal blood and/or surrounding tissues may directly contact fetal cells (**Figure 1A**). On the other hand, by mid second trimester and through term, barriers between fetal and maternal blood are reduced to fetal endothelium, STB and a discontinuous CTB (Moalli et al., 2011). Here, fetal villous trees are fully bathed in maternal blood. This critical difference in placental architecture is represented in **Figures 1B, C**.

## 3 Cell derived models to study *T. gondii* in the human placenta

### 3.1 Immortalized cell lines

Cancer-derived and *in vitro* immortalized trophoblastic cells have been traditionally used to model placenta. These cell lines are easily obtained and manipulated, but they have abnormal karyotypes and altered gene expression, which may not faithfully represent trophoblast *in vivo* behavior (Apps et al., 2009; Novakovic et al., 2011; Kallol et al., 2018). By far, the most widely used trophoblast cell line is BeWo. BeWo cells are choriocarcinoma derived and originally developed as a cancer research model and for the *in vitro* production of human chorionic gonadotropin (hCG) (Hart et al., 1968; Pattillo et al., 1971). BeWo have been extensively used in *T. gondii* research to study infection in the context of the maternal fetal interface. In this cell line, *T. gondii* concentrates around intercellular junctions and regulates host´s ICAM-1 (Intercellular Adhesion Molecule 1), suggesting that the parasite exploits the paracellular route for invasion (Barragan et al., 2005; Pfaff et al., 2005a). Infections in BeWo showed that these cells are more susceptible to *T. gondii* than HeLa cells (uterine cervical tumor derived). Consistently, both cell lines produce different immune effectors in response to infection (Pfaff et al., 2005b; Oliveira et al., 2006).Additionally, ICAM-1 expression in both cell lines is differentially induced by TGF-β1 and IFN-γ, suggesting a different modulation of susceptibility to infection(Teixeira et al., 2021). Another interesting finding is the parasite’s ability to modulate apoptosis as an evasion strategy to survive. This has been observed in a broad range of trophoblast models, including BeWo (Angeloni et al., 2009), JEG-3 (Wei et al., 2018), HTR8/SVneo (Guirelli et al., 2015), isolated primary trophoblasts (Liu et al., 2013), and additionally, a human monocyte cell line, THP-1 (da Silva Castro et al., 2021).

As mentioned, one particularly susceptible moment for *T. gondii* to meet fetal trophoblast is during EVT invasion of placental formation. Experiments in immortalized EVT (HTR8/SVneo) (Graham et al., 1993) indicate that this type of trophoblast is highly susceptible to *T. gondii’s* infection (Milian et al., 2019; Ye et al., 2020).

Classical immune response to *T. gondii* infection entails a pro-inflammatory response, with the production of multiple cytokines and immune effectors, including IL-6, IL-12, IL-10, (TNF)-α, interleukin (IL)-1β and IFN-γ, among many others. Macrophage migration inhibitory factor (MIF) is a pro-inflammatory factor needed to control *T. gondii* infection (Flores et al., 2008), playing a pivotal role in the control of the infection particularly during gestation. MIF’s differential expression among first and third trimester placental explants (De Oliveira Gomes et al., 2011) has been linked to the higher susceptibility to congenital infection of the third trimester. Trophoblast models have been instrumental in deciphering cell-type specific routes of immune modulation elicited by *T. gondii* infection. EVTs display higher levels of MIF, its receptor, CD74, and co-receptor, CD44, than CTB. *T. gondii* infection further induces MIF production in EVTs. Surprisingly, MIF pharmacological inhibition in EVT leads to a significant decrease in *T. gondii*´s proliferation. In contrast, addition of recombinant MIF (rMIF) to infected EVTs, leads to increased CD44 co-receptor expression, ERK1/2 phosphorylation, COX-2 expression, and IL-8 production, all of which seem to favor *T. gondii*´s proliferation (Milian et al., 2019). On the other hand, BeWo cells naturally exhibit reduced expression of MIF, and this has been associated with higher susceptibility to infection by *T. gondii* (De Oliveira Gomes et al., 2011; Milian et al., 2019).

Trophoblast models have also served in demonstrating that *T. gondii* down-modulates the production of IL-6 and MIF by ways of inducing cyclooxygenase (COX-2) and prostaglandin E2 (PGE2) production. Lipid droplets are known sites of production and accumulation of COX-2. Consistently, it was observed that *T. gondii* induces an increase in lipid droplets in both BeWo and HTR-8/SVneo cells (de Souza et al., 2021).

Heme Oxygenase 1 (HO-1) activity controls parasite replication, and the expression is particularly diminished in EVT, which is also more susceptible to infection than CTB. This observation is supported by the differential expression of this enzyme in the immortalized models HTR8/SVneo compared to BeWo (Almeida et al., 2021) and their primary culture equivalents (Bilban et al., 2009).

### 3.2 Placental models derived from primary cells

Primary cells are cells that have been isolated from a tissue of a multicellular organism. This type of culture is often restricted in terms of the number of viable passages, and more demanding of particular growth conditions and supplements. At the same time, primary cells provide a more representative platform to work as they are genetically stable and retain the functional and morphological characteristics of their tissue of origin. In the following sections, we will review primary cell models used to study host-*T. gondii* interactions, following the logic of placental architecture from the maternal myometrium to the fetal capillaries, recapitulating the subsequent tissue layers that parasites must cross in order to reach the new individual.

#### 3.2.1 Decidual cells

The decidua refers to the gravid endometrium. The decidua basalis (db) is the particular endometrial portion that eventually forms the placenta. The db becomes separated from the uterus after parturition. The decidua controls trophoblast invasion through hormonal production (Moore et al., 2020). In addition, during the process of decidualization, endometrial resident cells acquire specific characteristics to serve as a rich source of nutrition for the embryo. Another important function of decidual resident cells is to set up the regulatory tolerogenic, yet immune active, state needed for the fetus to thrive (Van Der Zwan et al., 2017). These special features may not be present in counterpart cells residing in other tissues. Primary decidual cells can be obtained from full term placenta db tissue, and diverse cell types can be recognized based on expression patterns of specific marker.

Decidual fibroblasts (Ander et al., 2018) and dNKs (Zhang et al., 2015) are highly permissive to infection by *T. gondii*, and their response to infection is related to TB apoptosis and subsequent damage to the placental barrier. In *T. gondii*-infected primary decidual macrophages, different molecular pathways are activated biasing their differentiation towards an M1 phenotype, thus weakening their M2 tolerance function (Li et al., 2017; Zhang et al., 2019), which is paramount to a healthy pregnancy. Decidual dendritic cells, key players in the maintenance of the tolerogenic state of the placenta, are also induced to a dysfunctional phenotype during *T. gondii* infection (Sun et al., 2022). On the other hand, different immune cells acquire a highly migratory phenotype after they get infected (Ueno et al., 2015; Ólafsson and Barragan, 2020), and they do so without stimulating immune responses (Courret et al., 2006; Lambert et al., 2006; Hoeve et al., 2022), all of which is beneficial for *T. gondii’s* dissemination. Evidence regarding this trojan horse phenomenon has been obtained from measures of the migration patterns of *in vitro* infected bone-marrow derived DCs in a BeWo-coated transwell system, from infections in pregnant mice and in human PBMCs derived from peripheral blood (Lambert et al., 2006; Lambert et al., 2009; Collantes-Fernandez et al., 2012; Hoeve et al., 2022). To our knowledge, the migratory phenotype and trojan horse strategy has not been observed yet in human decidual cells.

#### 3.2.2 Trophoblast cells

Primary human trophoblasts (PHT) can be obtained from fresh placental tissue through enzymatic dispersion and immunomagnetic purification (Salomon et al., 2015). Purified CTBs have proliferative capacity and, with the addition of epidermal growth factor (EGF), the cells can undergo robust differentiation forming STB-like cells. It has been shown that CTB and STB obtained from primary cultures can be readily infected with *T. gondii*, protecting them from apoptosis, except when co-cultured with Interferon gamma producing dNKs (Abbasi et al., 2003; Zhang et al., 2015). On the other hand, STBs are less susceptible to *T. gondii* attachment and replication compared with primary CTBs and trophoblast cell lines (BeWo, JEG-3) (Ander et al., 2018). Please note that *T. gondii’s* infection has been assayed for an array of intermediate host-derived trophoblasts. These include, but are not limited to, mice (Wang et al, 2018) and sheep (Fernández-Escobar et al, 2021). Varying results regarding infectivity have been obtained, likely reflecting host-specie and parasite-strain specific dynamics.

#### 3.2.3 Fetal endothelial cells

As transplacental passage of *T. gondii* may occur by migration across epithelial/endothelial barriers, endothelial cells are relevant models to take into consideration when studying vertical transmission.

There are two types of endothelial cells that form the placenta vasculature. The human placental microvascular endothelial cells (HPMECs), present in the fetal capillaries of chorionic villi, and the macrovascular human umbilical vein endothelial cells (HUVECs). The first ones are obtained from the distal side of the human placenta, and purified by magnetic isolation of CD31 marker (Huang et al., 2018). As for the HUVEC cells, they are obtained from the umbilical cord vein by collagenase digestion (Siow, 2012). These endothelial cells differ in morphology and function (Lang et al., 2003). Particularly, HPMECs have higher responses to FGF2, VEGF and EG-VEGF, factors that promote angiogenesis (Huang et al., 2018). Regarding *T. gondii*, it has been reported that HUVECs and HMEC-1 (a stable cell line from dermal human microvasculature) present different infection susceptibility to two *T. gondii* strains (ME49 and RH) in a cell type/parasite combination dependent fashion (Cañedo-Solares et al., 2013). HUVECs cells have also been used to demonstrate that *T. gondii* induces the remodeling of the endothelial cytoskeleton and alteration of the cell barrier function (Franklin-Murray et al., 2020).In addition, infection of bovine derived vein endothelial cells (BUVECs) displays altered progression through the cell cycle (Velásquez et al., 2019), with increased host cell proliferation and an enhanced number of multinucleated cells. HUVEC are also frequently applied in the development of more complex placental models, resembling the fetal compartment, from 2D co cultures (Wong et al., 2020), to organ-on-a-chip systems (Lee et al., 2016).

#### 3.2.4 Fetal macrophages

Other immune cells that are highly abundant in the human placenta are the fetal-origin macrophages called Hofbauer cells (HBCs). These cells are thought to play an important role in protecting the fetus from vertical infections and to influence trophoblast and placental vascular development (Thomas et al., 2020; Fakonti et al., 2022). To our knowledge, there are no reports of HBC responses to *T. gondii* infections. Nonetheless, observational studies of another apicomplexan parasite, *Plasmodium falciparum* determined a subtle decrease in anti-inflammatory M2 percentage of HBCs in infected placentas from primigravidas. Most importantly, this study determined this phenotype to be highly predictive of decreased fetal body weight, suggesting a protective effect of M2-type HBCs on fetal growth (Gaw et al., 2019). As a similar shift towards M1 phenotype has been reported for decidual macrophages when infected with *T. gondii *(Zhang et al., 2019), it would be interesting to evaluate HBCs' phenotypes in this condition.

### 3.3 Stem cell derived models

Primary cultures display several advantages over immortalized cell lines. Because they are derived directly from tissue and not genetically modified, they usually retain many of the differentiated characteristics of the cell *in vivo*, providing excellent models for studying normal physiology and cellular metabolism. However, they can be arduous to obtain, have a finite lifespan and a limited expansion capacity, making it difficult to sustainably work with them. An alternative to primary culture is the use of stem cells, which are a reproducible, natural and renewable source of cells. Stem cells can be differentiated into diverse cell types under defined culture conditions (Snykers et al., 2009; Mummery et al., 2012; Kim et al., 2016).

#### 3.3.1 Mesenchymal stem cell-derived models

One source of fetal cell models used to study congenital transmissions are the mesenchymal stem cells isolated from human umbilical cord. During infection with *T. gondii* these cells are induced towards autophagic cell death by a mechanism that involves downregulation of mitochondrial stress factor Mcl-1 (Chu et al., 2017).

#### 3.3.2 Trophoblast stem cells

Okae and collaborators have reported the derivation of human trophoblast stem cells (hTSC) from CTB and blastocysts. These cell lines were further able to differentiate in CTB, STB and EVT, and showed transcriptomes similar to primary trophoblast cells meeting the criteria for human trophoblast cells proposed by Lee and collaborators (Lee et al., 2016; Okae et al., 2018). Another putative hTSC line is the USFB6, obtained from an eight-cells human morula. These cells have a more mesenchymal-like morphology than the TSC population isolated by Okae. However, trophoblast criteria have not been completely determined (Zdravkovic et al., 2015). Some differentiation protocols manage to accurately recapitulate hallmarks of TB including syncytialization and migration (Gerami-Naini et al., 2004; Castel et al., 2020). Trophoblast-like cells can also be obtained by differentiation of human embryonic stem cells (hESC) and induced pluripotent stem cells (iPSC). The most common approach to experimentally induce hESC differentiation towards trophoblast-like cells is BMP4 treatment. However, differentiation in this model system is difficult to control, as other cell types (mesodermal and endothelial cells) also appear in the culture, protocols are highly variable, and it is not clear to what extent they accurately mimic real TSCs (Gamage et al., 2016).

#### 3.3.3 Trophoblast organoids

Trophoblast organoids are an additional promising cellular model derived from stem cells. These long-term expanding cellular structures, can be developed from first trimester placental villi (Haider et al., 2018; Turco et al., 2018) or TSC derived from hIPSC (Karvas et al., 2022). These cultures organize into villous-like structures, and recapitulate differentiated subtypes of TB (CTB, EVT and STB), adding 3D orientation. Though, to our knowledge, trophoblast organoids have not been used to study *T. gondii*´s infection, recently, TSC-derived organoids have shown to recapitulate placental viral infectivity to Zika and SARS-CoV-2 virus (Karvas et al., 2022). These findings reinforce the relevance of trophoblast organoid models for studying other pathogens implicated in adverse pregnancy outcomes.

### 3.4 Human placental explants

Higher levels of model complexity have been achieved through the use of material from embryos and placentas from spontaneous or voluntary abortions. As mentioned before, TSCs derived from blastocysts have the ability to differentiate into different types of functional CTB, STB and EVT (Okae et al., 2018). Placental explants are an alternative source of all of these cell types. Robbins and collaborators isolated chorionic villi trees from placentas of 4-8 weeks of gestational age and reproduced the villous region and the EVT which invades uterine decidua. Their results indicate that it is the EVTs that are more susceptible to *T. gondii* infection (Robbins et al., 2012). In all cases, access to this material is limited and dependent on local legislation.

However, given the material is available, isolation of HPE is a simple procedure. If the appropriate culture conditions are provided, placental cells can be cultured for up to 5 days, maintaining tissue architecture and viability. Additionally, HPE represents a platform to study STBs which cannot be isolated because of their syncytial nature. STB resistance to attachment of *T. gondii* was also observed in second-trimester chorionic villous explants. Interestingly, transcriptional analysis showed that only 22 out of 172 genes are similarly induced between infected explants and infected isolated primary TBs (Ander et al., 2018), highlighting the importance of tissue architectural context in cellular responses. MIF is upregulated with *T. gondii* infection in first trimester HPE and results in increased monocyte adhesion (THP-1 cells) to fetal villi, possibly facilitating pathogen transfer across the placental barrier (Ferro et al., 2008). Differences in the induction of MIF are found to be gestational age dependent as it is upregulated in first-trimester HPE but not in third-trimester HPE(De Oliveira Gomes et al., 2011). These findings, together with differences in frequencies of congenital toxoplasmosis according to gestational age, may point towards the use of distinct mechanisms of transplacental passage by *T gondii*. While migration in infected macrophages may be exploited during the first trimester, extracellular passage could be happening in full term placenta whereby cellular barriers are weakened.

Kremmerling and collaborators compared the infectivity of *T. gondii* and *T. cruzi* in explants derived from human, canine and ovine full-term placentas. Their findings indicate that in all scenarios *T. gondii* invades more efficiently and induces more tissue damage than *T. cruzi* (Liempi et al., 2020). On the contrary, when zooming in on the molecular alterations of placenta upon infection, the same group showed that in HPE a stronger pro-inflammatory response occurs during *T. cruzi* infection when compared to *T. gondii*. Additionally, parasites stimulate distinct repertoires of immune response mediators, TLRs, cytokines, and signaling pathways (Castillo et al., 2017; Liempi et al., 2019). Authors correlate these findings to the fact that vertical transmission of Chagas disease is less frequent than vertical transmission of toxoplasmosis(Castillo et al., 2017; Liempi et al., 2019). The association of immunological silence and a more successful transplacental passage has been described for *T. cruzi* isolates with a history of transgenerational congenital transmission, in a murine vertical transmission model (Faral-Tello et al., 2022). Immune response silencing of the placenta could also underlie in part *T. gondii*’s success in vertical transmission, though this hasn’t been experimentally addressed.

### 3.5 Other placental 3D models

Placental models have been improved with the advent of technologies that allow the generation of three-dimensional (3D) cultures. As mentioned, in the 3D context, the biological environment is better recreated allowing more relevant results at the anatomical and physiological level (Antoni et al., 2015). Among three dimensional systems, spheroids (Fennema et al., 2013) are the simpler ones. They can be technically constructed in two ways: taking advantage of the natural abilities of some cell types to aggregate and self-assemble into spherical structures, or by giving the culture a biocompatible spheroidal support such as hydrogel or collagen (Ryu et al., 2019). In this way, these multicellular structures can recreate the original cell-cell and cell-matrix junctions, key structures to study host-pathogen interactions.

Spheroids have contributed to recreating crucial stages of the life cycle of some parasites that were not being fully modeled in conventional cultures. For example, the reconstruction of the complete cycle, including the *in vitro* reactivation, of the *Plasmodium falciparum* in hepatocytes was achieved using this model (Chua et al., 2019). Novel mechanisms of *T. cruzi* migration through the paracellular route were observed using spheroids (Jones et al., 2017). Fundamental results for more complete understanding of the phenomena of mobility, migration, replication, egress and development of the sexual stages of *T. gondii* were only achievable *in vitro* by applying three-dimensionality (Ramírez-Flores et al., 2022). Moreover, spheroids have been used to recreate a complex placental process like trophoblast invasion (Wong et al., 2019), contributing substantially to understanding processes at the maternal-fetal interface. Spontaneous syncytialization (STB formation) of TB was only accomplished by 3D culture of JEG-3 cell line. This model allowed mimicking STB resistance to *T. gondii* when co cultured with human microvascular endothelial cells in a bioreactor 3D system (McConkey et al., 2016). This resistance phenotype was previously observed only in *ex vivo* infections of first trimester HPE (Robbins et al., 2012).

Advantages regarding the culture of immortalized cell lines enable the development of more complex 3D systems. Recently, BeWo cells were used for the construction of a placenta-inspired 3D bioprinted barrier model. Through the co-culture of TB (BeWo), placental fibroblasts (simulating placental stroma) and endothelial cells, authors were able to mimic the barrier that separates maternal blood from fetal blood in the full term human placental villous, achieving two weeks stability of the culture, without the use of an artificial membrane filter (Kreuder et al., 2020).

Organs-on-a-chip, which are 3D microfluidic devices that involve different cells to simulate activities, mechanics and physiological responses of an entire organ, have already been constructed to mimic the placenta (Blundell et al., 2016; Lee et al., 2016; Arumugasaamy et al., 2018; Nishiguchi et al., 2019; Yin et al., 2019). Most of these placenta-on-a-chip systems have been constructed using immortalized trophoblastic cell lines, BeWo and others. An exception is the work by Nishiguchi and collaborators, who used primary CTBs isolated from first and third trimester chorionic villi to this end (Nishiguchi et al., 2019). To our knowledge, microfluidic systems have neither been used to study *T. gondii*’s infection process nor host-pathogen interactions. However, work by Arumugasaamy and collaborators achieved productive experimental infections using Zika virus (Arumugasaamy et al., 2018) and Zhu and collaborators evaluated the inflammatory response of fetal (endothelial) and maternal cells (BeWo) to *E. coli*, incorporating THP-1 cells in the fluidic system (Qin et al., 2018). These models bear a great potential to study the biology underlying transplacental passage of pathogens, while also enabling the search for potential therapeutics directed to treat women`s chronic conditions during gestation, instead of the currently used strategy of suppressing medication, an area that has long been neglected in medical research (Couzin-Frankel, 2022).

## 4 Discussion: Challenges and opportunities for modeling *T. gondii*´s vertical transmission

The first difficulty in studying congenital transmission of *T. gondii in vitro* is faithfully modeling placental tissue complexity. Although hypotheses of transplacental passage have been formulated based on other models, mechanisms of parasitism occurring at the maternal-fetal interface have traditionally been out of reach to researchers because of the lack of accurate models.

The placenta has a complex cellular structure which varies greatly along gestation, and among species (Furukawa et al., 2014). Therefore, results obtained in animal models do not necessarily reproduce what happens in humans. In this sense, the development of different human cell lines and a variety of primary cultures obtained from human placentas have allowed us to approach specific biological phenomena. Significant steps forward have been possible, impacting our understanding of infection susceptibility of different cell types, signaling mechanisms triggered during invasion, immune responses and manipulation. Models have also provided platforms for testing antiparasitic drugs (for more details, see **Table 1**). Nonetheless, these cellular models, though immensely instrumental to a number of biological questions, pose limitations to our ability to fully recreate the *in vivo* biology.

**Table 1:** *In vitro* models of human placenta for studying the biology of *Toxoplasma gondii*.

Cell model	Name and reference	Source	Representative cell type	*T. gondii* associated studies and references
Cancer cell line 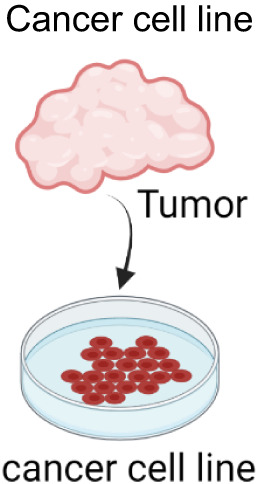	BeWo (Pattillo et al., 1968)	Choriocarcinoma explant	CTB/STB	Membrane adhesion(Teixeira et al., 2021); Infectionsusceptibility; (Almeida et al., 2019); Apoptosis modulation (da Silva Castro et al., 2021); Antiparasitic drugs (Ietta et al., 2017; Costa et al., 2021); Immune response (Castro et al., 2013).
	JEG-3 (Kohler and Bridson, 1971)	Choriocarcinoma explant	STB	2D and 3D infections (McConkey et al., 2016); Host apoptosis and RE stress (Wei et al., 2018).
	JAR (Pattillo et al., 1971)	Gestational choriocarcinoma	CTB	Infection and replication (Ander et al., 2018).
Immortalized cell line 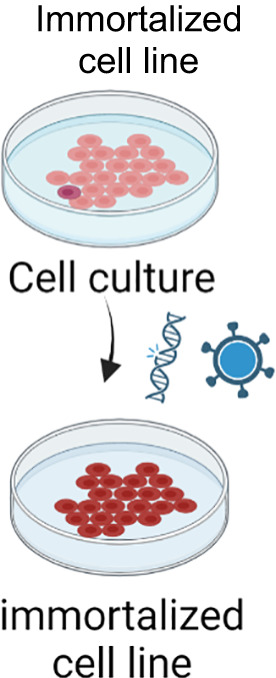	TCL-1 (Lewis et al., 1996)	Chorionic membrane	EVT	N/D
	ACH3P (Hiden et al., 2007)	Choriocarcinoma and first trimester trophoblast	CTB and EVT	N/D
	HPT-8 (Zhang et al., 2011)	First trimester placenta	EVT	N/D
	Swan 71 (Straszewski-Chavez et al., 2009)	First trimester placenta	CTB	N/D
	HTR-8/SVneo(Graham et al., 1993)	First trimester villous explant	EVT/CTB	Susceptibility to infection (Almeida et al., 2019); Modulation of cell death (da Silva Castro et al., 2021); Intracellular proliferation signaling (Milian et al., 2019); Antiparasitic treatment (Costa et al., 2021).
Primary cell 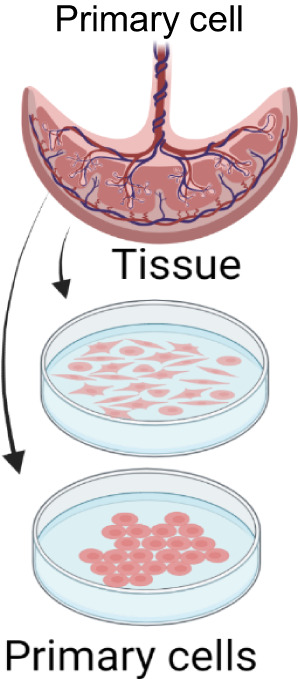	PHT (Human trophoblast cells)	Placenta	CYT and STB	Invasion, attachmentand replication (Abbasi et al., 2003; Ander et al., 2018)
	Decidual-derived cells	Decidua basalis	NK, fibroblast, macrophages, dDC	Invasion and susceptibility (Zhang et al., 2015; Ander et al., 2018); M1 and M2 phenotype switch (Li et al., 2017); Dysfunction of dDC(Sun et al., 2022).
	HUVEC (Jaffe et al., 1973)	Umbilical cord vein	Venous endothelial cells	Barrier function dysregulation (Franklin-Murray et al., 2020); Endothelial invasion (Cañedo-Solares et al., 2013).
Stem cell 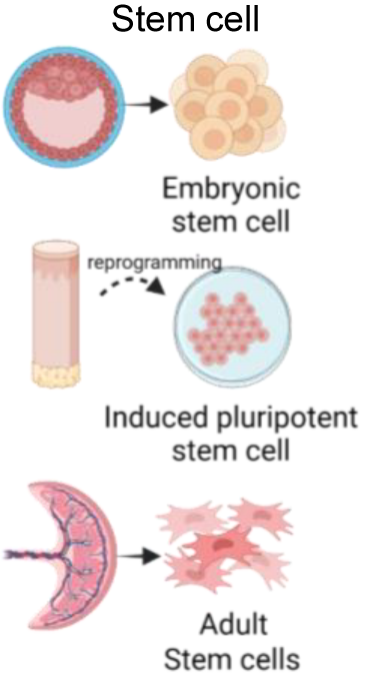	hTSC (Okae et al., 2018)	Blastocist/first trimester placenta	Trophoblast stem cells	N/D
	iTP (Chen et al., 2013)	Human fetal fibroblast	Trophoblast progenitor cells	N/D
	hUC-MSC (Chu et al., 2017)	Umbilical cord mesenchymal stem cells	mesenchymal stem cells	Host cell autophagy and apoptosis (Chu et al., 2017)
	hPSC-TS (Mischler et al., 2021)	Differentiated hESC or hiPSC	Trophoblast stem cells	N/D
	Organoids (Haider et al., 2018; Turco et al., 2018)	Stem cell/Villous tissue from first trimester placenta	trophoblast stem cells, CTB STB and EVT	N/D

*N/D, non-determined.

Importantly, cellular models usually rely on one or two different cell types, which cannot recreate the complex multicellular architecture of the original tissue. These limitations are solved, at least partly, by HPE, in which the structure, cellular diversity and interactions of the original tissue are better maintained, allowing placenta modeling closer to reality. Nevertheless, as a human primary culture, HPEs (obtained from term placenta or abortions) also harbor some challenges, especially regarding accessibility, reproducibility and maintenance, making it difficult to sustainably work with them.

Additionally, explants plated on culture dishes likely poorly mimic the characteristics of *in vivo* contact with parasites. In particular, parasitic load, and the way parasites access the villi are likely altered. For example, parasites firstly contacting the fetal part of the villous explants, something that would not occur *in situ* given the anatomy of the placenta, cannot be avoided.

Material from first trimester placentas has shown great potential in modeling different types of cells and placental processes. Access to these samples could be possible in countries where voluntary interruption of pregnancy is legal. However, the use of this material for research purposes has ethical constraints including specific medical procedures and coordinated efforts of the scientific and medical community. In the last years, the advances on stem cell technology have allowed scientists to surpass some of these limitations, enabling the establishment of more physiologically relevant *in vitro* cellular models, namely developing trophoblast organoids, in which genetically stable stem cells give rise to 3D cellular structures, resembling various aspects of the original tissue. Even when new challenges such as reproducibility, cellular differentiation degree, long-term culture maintenance, and 3D analytical tools development must still be overcome, the achievements made up to now indicate that we are on the right track.

It is fair to envision that these cellular models, coupled with bio-printed or organ-on-a-chip technology, will enable the development of more complex systems, integrating other cellular components (immune, stromal, endothelial cells) and fluidic forces. These improvements will allow scientists to delve deeper into how *T. gondii* invades fetal cells from maternal tissue, if there is a cell-type tropism for the parasite at the placenta or if there is a particular stage in the invasion process that could be used as a target for new drug development, contributing to shed light on the -so far- hidden mechanisms of *T. gondii* vertical transmission.

## Author contributions

PF-T and MF conceived this manuscript. RP and PF-T created the Figure and Table. MF and MB-F contributed to funding acquisition. All authors contributed to the article and approved the submitted version.
